# Toward explainable AI in radiology: Ensemble-CAM for effective thoracic disease localization in chest X-ray images using weak supervised learning

**DOI:** 10.3389/fdata.2024.1366415

**Published:** 2024-05-02

**Authors:** Muhammad Aasem, Muhammad Javed Iqbal

**Affiliations:** Department of Computer Science, University of Engineering and Technology, Taxila, Pakistan

**Keywords:** explainable artificial intelligence, class activation maps, weak supervised learning, computer aided diagnosis, ensemble learning, transfer learning

## Abstract

Chest X-ray (CXR) imaging is widely employed by radiologists to diagnose thoracic diseases. Recently, many deep learning techniques have been proposed as computer-aided diagnostic (CAD) tools to assist radiologists in minimizing the risk of incorrect diagnosis. From an application perspective, these models have exhibited two major challenges: (1) They require large volumes of annotated data at the training stage and (2) They lack explainable factors to justify their outcomes at the prediction stage. In the present study, we developed a class activation mapping (CAM)-based ensemble model, called Ensemble-CAM, to address both of these challenges via weakly supervised learning by employing explainable AI (XAI) functions. Ensemble-CAM utilizes class labels to predict the location of disease in association with interpretable features. The proposed work leverages ensemble and transfer learning with class activation functions to achieve three objectives: (1) minimizing the dependency on strongly annotated data when locating thoracic diseases, (2) enhancing confidence in predicted outcomes by visualizing their interpretable features, and (3) optimizing cumulative performance via fusion functions. Ensemble-CAM was trained on three CXR image datasets and evaluated through qualitative and quantitative measures via heatmaps and Jaccard indices. The results reflect the enhanced performance and reliability in comparison to existing standalone and ensembled models.

## 1 Introduction

The healthcare industry plays a pivotal role in ensuring the wellbeing of individuals and communities. Despite the rapid advancements in technology, most of the industry still relies heavily on manual procedures including, but not limited, to diagnosis and treatments. These manual procedures can be time-consuming and prone to errors in the result of workload and lack of facilities. Such factors may further lead to serious consequences such as misdiagnosis, incorrect treatment, and adverse patient outcomes (Silva et al., 2022). To overcome these challenges, various approaches have been explored to assist caregivers in decision-making by Computer Aided Diagnosis (CAD) (Doi, 2007). Among Fuzzy logic (Kovalerchuk et al., 1997), rule-based (Ion et al., 2009), and other predictive models (Yanase and Triantaphyllou, 2019), machine learning (ML) established outstanding potentials for CAD systems (Reyes et al., 2020). The most highlighted approach in machine learning is known as deep learning (DL) for its ability to learn complex and meaningful patterns from large volume of data (LeCun et al., 2015; Voulodimos et al., 2018; Shrestha and Mahmood, 2019; Georgiou et al., 2020; Mahony et al., 2020). In spite of its success in disease classification and localization, there are many internal and external challenges in deep learning (Aasem et al., 2022). Internal challenges include appropriate selection of hyperparameters and interpretability. Similarly, external challenges necessitate addressing the demands for high computational resources and large volume of training data.

Advancements in hardware technology, such as graphics processing unit (GPU), tensor processing unit (TPU), and application-specific integrated circuit (ASIC), have sufficiently addressed the demand of high computational need for deep learning (Mittal and Vaishay, 2019; Hu et al., 2022; Nikolić et al., 2022). However, acquisition of large volume data with task-specific annotation is still a challenge (Aasem et al., 2022). This becomes even more harder when annotation requires specialized skills and experience of radiologists. This study exploits weak supervised learning for dealing with the annotations issue for disease localization in chest X-ray images using deep learning. In general, X-ray images are examined by radiologists who specialize in the interpretation of similar reports related to diagnoses of chest, lungs, heart, and related disorders. In routine tasks, they can identify the patterns of related disorder just by visual examination. In some cases, multiple radiologists are engaged to discuss a given report for its complexity and criticality (Siegel, 2019). Such cases may not be concluded easily and may float with misperceptions. To resolve such cases, majority of votes, senior opinion weightage, or further testing are considered. Moreover, conclusive inferences are still made in conjunction with additional information such as patient history and current condition (Prevedello et al., 2019). This complexity makes the annotation process harder to accomplish for a large volume of images. This study discusses an indirect approach for localization, thereby aiming to overcome such dependency issues in weak supervised learning.

**Figure 1:**
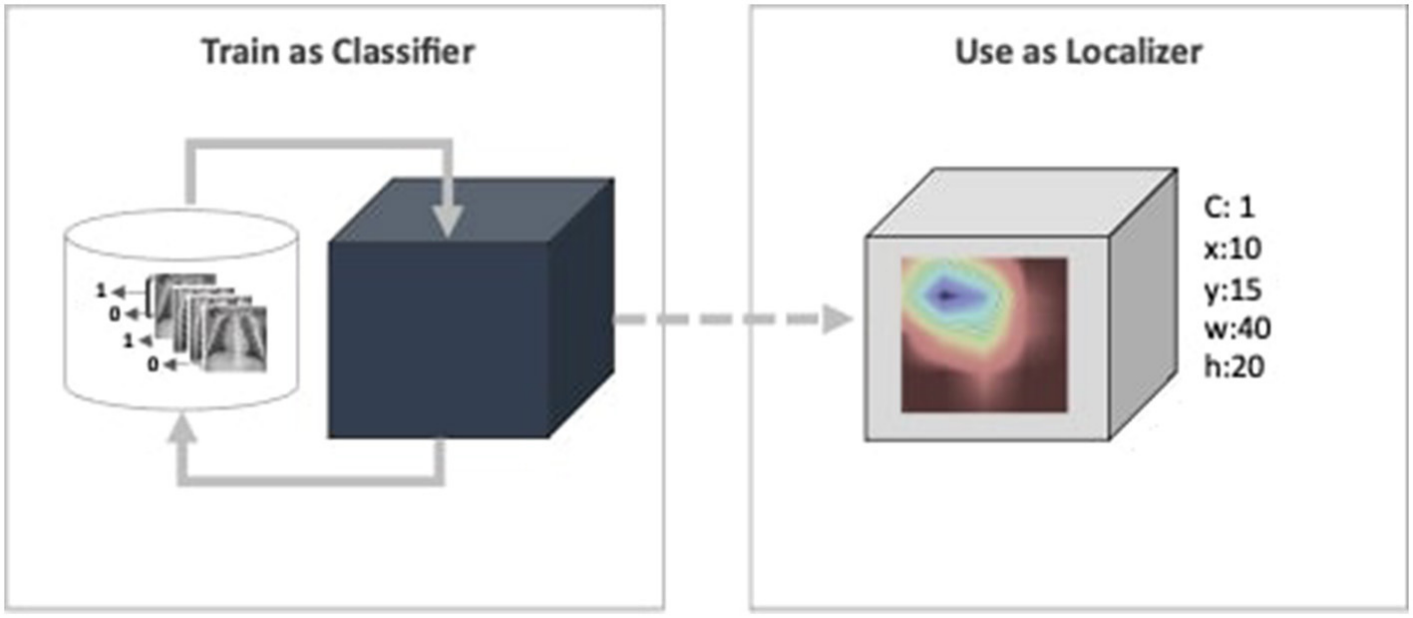
Extracting localization details via classification skills.

Furthermore, deep learning models have been deemed untrustworthy due to their non-justified inferences (Adabi and Berrada, 2018; Sheu and Pardeshi, 2022). Such behavior is critical for the CAD system that creates a major bottleneck for their practical application in the healthcare industry (Reyes et al., 2020; Elhalawani and Mak, 2021; Yu et al., 2022; Park et al., 2023). Despite overlooking the need for the model's self-justification concern, they are evaluated based on their performance metrics for given datasets. As highlighted by Wagstaff (2012), models must be measured beyond benchmarked datasets and quantitative metrics. Predicting a medical image as positive or negative disorder does not answer completely from the radiologist's perspective. “How the prediction inferred?" is also a matter interest of transparency and reliability view points (Adabi and Berrada, 2018). To address the transparency concern, the proposed work aims to employee CAM as function. The existing literature have discussed CAM and its variants for single model interpretability within the limited scope, i.e., visual evaluation. The proposed framework is referred to as Ensemble-CAM because it extends the current scope in two directions: First, it allows multiple models in the ensemble learning paradigm to generate a single set of interpretable features. Second, it evaluates the intermediate and final outcomes using quantitative metrics, i.e., Jaccard index or Intersection over Union (IoU). An intuitive illustration of the proposed framework has been illustrated in Figure 1. This depicts a weakly supervised pipeline, where the image classifier is trained on X-ray images in the first phase. Until this phase, the model is a black box, capable only of predicting a class value. The next block consists of a CAM function that generates a heatmap and reveals activated features. The heatmap further constitutes spatial information in the form of bounding box coordinates.

The rest of the study is organized into four main sections. In Section 2, a brief overview of related literature is provided, serving as a foundation for the proposed methodology outlined in Section 3. This methodology includes details on the dataset utilized, the proposed technique, and the experimental methodology employed. The results and discussions are presented in Section 4, providing insights into the outcomes of the study. Finally, in Section 5, the study concludes with a comprehensive summary of the findings and directions for future research, offering a glimpse into the potential avenues for growth and advancement in this field.

## 2 Literature review

Deep learning has revolutionized computer-aided diagnosis (CAD) in medical imaging, marking significant progress since the last decade (Ma et al., 2021). Its successful integration into various medical fields, particularly in radiology (Reyes et al., 2020; Chandola et al., 2021), dermatology (Esteva et al., 2017; Rezvantalab et al., 2018; Jeong et al., 2022), and cardiology, demonstrates its versatility and effectiveness. In radiology, deep learning models such as DenseNet (ea Shortliffe, 1975) and ResNet have been instrumental in enhancing the detection and diagnosis of abnormalities in chest X-ray images, evolving from traditional rule-based methods to more advanced, reliable solutions (Doi, 2007). These models have not only improved diagnostic accuracy but also introduced flexibility, making them adaptable across different imaging modalities. Despite their success, these deep learning approaches face challenges such as data dependency and interpretability, necessitating a balanced evaluation of their impact on medical imaging and patient care.

Explainable AI (XAI) techniques in medical imaging have gained traction for enhancing the transparency and trustworthiness of deep learning models (Giuste et al., 2023). Tools, such as Grad-CAM, Yan et al. (2018) and Guan et al. (2020) provide visual explanations of model decisions, particularly in chest X-ray analysis, by highlighting relevant areas influencing the diagnostic outcome. This advancement is crucial in radiology, where understanding the rationale behind AI predictions is as important as the predictions themselves. Shi et al. (2021) further emphasizes the role of XAI in combating pandemics, showcasing how these methods can bridge the trust gap in clinical decision-making during critical health crises. Although XAI has empowered radiologists with better interpretative insights, it still faces challenges, such as the potential for misinterpretation and the need for improved methods to accurately reflect the underlying model logic. The integration of XAI in medical imaging thus represents a pivotal step toward more reliable and interpretable diagnostic systems, fostering greater acceptance and confidence among medical professionals (Szegedy et al., 2013; Rao et al., 2020). Rani et al. (2022a) proposed model Covid-Scanner detects COVID-19 in chest radiographs through a multi-modal system. By combining bone suppression, lung segmentation, and classification they further utilize GradCAM++ for feature visualization.

Similarly, Caroprese et al. (2022) explores argumentation approaches in XAI, offering structured justifications for medical decisions, thereby improving explainability and transparency. Although XAI has empowered radiologists with better interpretative insights, it still faces challenges, such as the potential for misinterpretation and the need for improved methods to accurately reflect the underlying model logic. The integration of XAI in medical imaging, including argumentation theory, thus represents a pivotal step toward more reliable and interpretable diagnostic systems, fostering greater acceptance and confidence among medical professionals (Szegedy et al., 2013; Rao et al., 2020). The CovidScanner model (Rani et al., 2022a), for instance, detects COVID-19 in chest radiographs through a multi-modal system, utilizing GradCAM++ for feature visualization and exemplifying the practical application of XAI in pandemic response.

Weakly supervised learning has emerged as a promising approach in chest X-ray image analysis, addressing the scarcity of finely annotated medical images (Islam et al., 2017; Ouyang et al., 2020). Unlike strongly supervised methods that require detailed annotations, weak supervision leverages image-level labels to localize and identify pathological features, thereby mitigating the extensive effort and expertise needed for detailed labeling. Despite its cost-effectiveness and reduced annotation requirements, weakly supervised models often face challenges in achieving the high precision and specificity seen in fully supervised systems. The balance between model performance and the availability of limited annotated data is critical, making weakly supervised learning a key area of research for improving accessibility and efficiency in medical diagnostics (Rozenberg et al., 2020; Wehbe et al., 2021). This approach not only broadens the applicability of deep learning in resource-constrained settings but also encourages advancements in algorithmic efficiency and interpretability.

Table 1 presents an overview of abnormalities detection approaches for X-ray images. The comparative analysis of deep learning methods in medical imaging, especially in chest X-ray analysis, reveals a diverse landscape of methodologies ranging from traditional machine learning to advanced deep learning and weakly supervised models (Rajpurkar et al., 2017; An et al., 2022). Each method presents its own set of advantages and limitations. For instance, while deep learning models such as DenseNet and ResNet have shown remarkable success in accuracy and reliability, they require substantial data and computational resources (ea Shortliffe, 1975). The SFRM-GAN (Rani et al., 2022b) enhances bone suppression while preserving image quality and spatial resolution. On the other hand, weakly supervised approaches offer a solution to limited data scenarios but may compromise on localization precision (Ouyang et al., 2020). The critique of these methods underscores the need for a balanced approach that considers both the technical and practical aspects of medical image analysis. It emphasizes the importance of interpretability, resource efficiency, and adaptability to varying clinical needs, guiding future research Toward more holistic and context-aware diagnostic solutions (Yan et al., 2018; Ponomaryov et al., 2021).

**Table 1:** Summary of relevant approaches for detection of abnormalities in X-ray images.

**Refereces**	**Methodology**	**Ensembled**	**Interpretability**	**Localization**	**Evaluation**
Rajpurkar et al. (2017)	DenseNet-121	No	Grad-CAM	Heatmap	Visual
Islam et al. (2017)	ResNet-50, ResNet-101, ResNet-152	Yes	Convnet up-sample	Heatmap	Occlusion sensitivity
Rozenberg et al. (2020)	Specialized loss function, anti-aliasing filters, and conditional random field layers	No	No	No	IoU
An et al. (2022)	ResNet + channel attention	No	No	Channel attention	No
Yan et al. (2018)	DenseNet, squeeze-and-excitation block, multi-map transfer layer, max-min pooling operator	No	Grad-CAM++	Heatmap	Visual
Guan et al. (2020)	AG-CNN (Global block, Local block, Fusion)	No	Grad-CAM	Heatmap	Visual
Wehbe et al. (2021)	DeepCOVID-XR (DenseNet-121, ResNet-50, InceptionV3, Inception-ResNetV2, Xception, EfficientNet-B2)	Yes	Grad-CAM	Heatmap	Visual
Ouyang et al. (2020)	Foreground, positive, and abnormality attentions	No	Grad-CAM	BBox	IoU
Wu et al. (2020)	6-region-slice, U-Net	No	No	BBox	IoU
Ponomaryov et al. (2021)	X-Ray CAD (DenseNet-201, ResNet-50, EfficientNet)	Yes	Grad-CAM	Heatmap	Visual
Rani et al. (2022a)	Multi-modal bone suppression, lung segmentation	No	Grad-CAM++	Heatmap	Visual

The comparison of different methodologies in the literature.

Current trends in medical imaging, particularly in chest X-ray analysis, indicate a growing emphasis on addressing the challenges of labeled data acquisition, transparency, and reliability (Irvin et al., 2019; Wu et al., 2020). The acquisition of labeled data remains a significant bottleneck, with efforts such as CheXpert (Irvin et al., 2019) aiming to expand the availability of annotated datasets for training more robust models. Transparency in AI decisions is another critical aspect, where models such as U-Net and RetinaNet are being adapted to provide clearer insights into diagnostic decisions (Wu et al., 2020). However, the reliability of these AI systems, especially in the face of noisy or limited data, continues to be a concern (Rao et al., 2020; Szegedy et al., 2013). The end-goal is to develop AI systems that not only perform well under various constraints but also earn the trust of medical professionals through transparent and interpretable outputs. Addressing these challenges requires ongoing innovation in machine learning techniques and a deeper understanding of the clinical context, to ensure that the development of AI in medical imaging aligns with the real-world needs of healthcare providers and patients.

## 3 Materials and methods

**Figure 2:**
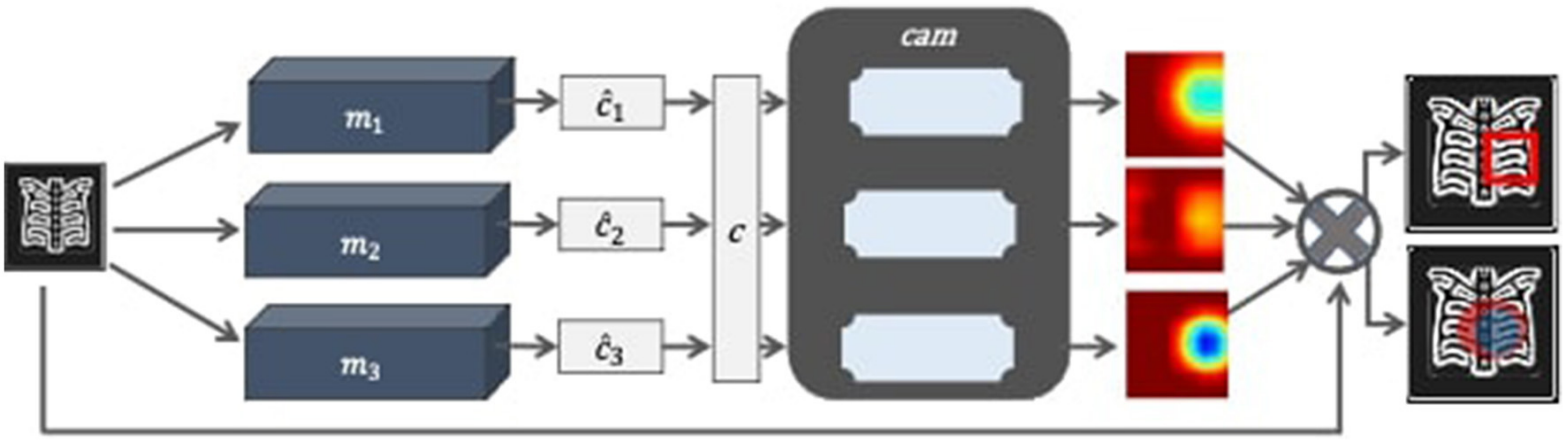
Block diagram of Ensemble-CAM for localizing abnormalities in the X-ray image with interpretable outcomes.

The proposed model consists of three main components, namely classification, class activated mapping, and aggregation. It also employs two supporting components that shall be referred as classfinalizer and heatmap-generator. The architecture of the proposed model follows ensemble learning at the classification and localization stages and is named as Ensemble-CAM. As illustrated in Figure 2, it requires no localization annotations at the training phase, yet capable to produce the bounding box and segmentation details in the explainable format. The output of Ensemble-CAM consists of aggregated class value, bounding boxes, mask, and heatmaps that interpret the result formation.

This section briefly explains the methodology of proposed work in detail. First, the sub-section describes the properties of datasets for the experiments while subsequently listing the deep learning classifiers. Next, conceptual definitions are established in general for class activation mapping and heatmap generation. Finally, Ensemble-CAM is defined and demonstrated via some test data.

### 3.1 Dataset

Three datasets have been considered to validate the performance of the proposed approach. To classify and localize pneumonia, the RSNA pneumonia detection dataset (Anouk Stein, 2018) has been used with 14,864 images to train the classifiers. In this dataset, 6,012 images have been marked positive for pneumonia, while 8,851 show no relative symptoms. For all pneumonia confirming images, the dataset also offers bounding box ground truth which was not used during the training phase. Similarly, the Chest-Xray-14 dataset (Wang and Peng, 2017) has been considered to detect cardiomegaly. The classifiers have been trained only for 9,628 images in which 4,000 images show enlarged hearth visuals. The dataset contains a small subset of images that have bounding box annotations which were ignored during training the classifier but considered in testing. The third dataset contains radiographs that have been tagged as COVID-19 confirming cases (Chowdhury et al., 2020; Rahman et al., 2021). Unlike the previous two datasets, there exist no bounding box annotations in this dataset. Therefore, a quantitative metric for localization has not been applied to demonstrate the model's performance. Table 2 shows the distribution of given datasets for training and validation during the training phase.

**Table 2:** Datasets for demonstration of Ensemble-CAM performance.

**DATASET**	**TARGET**	**TRAIN**	**VALID**	**TOTAL**
RSNA	Pneumonia	11,891	2,972	14,864
Chest X-Ray14	Cardiomegaly	5,477	1,369	6,846
COVID-19	COVID-19	7,703	1,925	9,628

### 3.2 Methods for evaluation

The performance evaluation metrics in this study has been split into two groups task-wise. For the classification task, accuracy (Equation 1), recall (Equation 2), and precision (Equation 3) have been computed. Similarly, Intersection over Union (Equations 4, 5) (also known as the Jaccard index) has been used to measure the quality of the localization task. The base components for all these metrics are as follows:

- True positive: output that correctly indicates the presence of a condition.
- True negative: output that correctly indicates the absence of a condition.
- False positive: output that wrongly indicates the presence of a condition.
- False negative: output that wrongly indicates the absence of a condition.

Accuracy: accuracy is a primary metric that refers to the ratio of number of correct predictions to the total number of input samples.


(1) $$\begin{array}{l} \text{Accuracy}=\frac{\text{number of correct predictions}}{\text{total number of predictions made}} \end{array}$$

Recall: recall is the proportion of actual positive cases that are correctly identified.


(2) $$\begin{array}{l} \text{Recall}=\frac{\text{true positive}}{\text{true positive}+\text{false negative}} \end{array}$$

Precision: precision also known as positive predictive value (PPV), refers to the proportion of positive cases that were correctly identified.


(3) $$\begin{array}{l} \text{Precision}=\frac{\text{true positive}}{\text{true positive}+\text{false positive}} \end{array}$$

Intersection-over-Union: the metric is well known for object detection task in strong supervised learning. It quantifies the degree of overlap between predicted and ground-truth boxes. Its values range from 0 to 1 where 0 refers to no overlap and 1 declares perfect overlap.


(4) $$\begin{array}{l} \text{IoU}=\frac{\text{area of overlap}}{\text{area of union}} \end{array}$$

In confusion matrices, it can be expressed as follows:


(5) $$\begin{array}{l} \text{IoU}=\frac{\text{TP}}{\text{TP}+\text{FP}+\text{FN}} \end{array}$$

The keynote for IoU in weak surprised learning is the unavailability of ground truth values. This makes it challenging to validate the performance of the given model. To quantify the proposed model performance with IoU, this study includes two datasets with bounding box annotated ground-truth. They have not been exposed during training but used at test instances only.

### 3.3 Ablation study for classification task

The ablation study conducted as part of this research aimed to evaluate a comprehensive range of deep learning image classifiers for the task of disease localization in chest X-ray images. Included in this assessment were AlexNet (Krizhevsky et al., 2017), VGG-16 & VGG-19 (Simonyan and Zisserman, 2014), ResNet-50 (He et al., 2016), EfficientNetB1 (Tan and Le, 2019), NasNetMobile (Zoph et al., 2018), MobileNetV2 (Sandler et al., 2018), DenseNet169 (Huang et al., 2017), and DenseNet121 (Huang et al., 2017).

The common hyperparameters employed in training these models are detailed in Table 3. The experiments were executed on a 64bit Ubuntu 20.04.5 LTS platform, powered by an Intel^®^Core i5-3470 CPU @ 3.20GHz x 4 and an NVIDIA GeForce GTX 1080 GPU, utilizing Python 3.9.12 with tensorflow 2.4.1 and keras-gpu 2.4.3.

**Table 3:** Configurations for training the image classifiers.

**Dataset**	**Key**	**Value**
Dataset	Split	Ratio: 70/30
	Color mode	RGB
Callback	Model checkpoint	Monitor: validation accuracy. Mode: Max
	Early stopping	Monitor: validation loss. Min_delta: 0.01. Patience: 6. Mode: auto. Baseline: None
	Reduce LR on plateau	Monitor: validation loss. Factor: 0.01. Patience: 4. Mode: auto. Min_delta:0.001
	others	TerminateOnNaN
Hyper-parameter	Max. Epoch	50
	Optimizer	Adam
	Loss	Categorical Crossentropy
	Initial weights	Imagenet
	Output layer	Softmax

The initial phase of this study revealed that the models with fewer layers, such as AlexNet, VGG-16, VGG-19, and NasNetMobile, did not perform optimally on the chest X-ray datasets, which characteristically exhibit less feature variation than other types of image datasets. Thus, these models were excluded from the subsequent training rounds. Deeper and more complex architectures were then subjected to a rigorous second round of training.

The subsequent evaluations led to the selection of DenseNet models and Xception for their exemplary performance metrics, while ResNet-50, InceptionV3, and MobileNetV2 were phased out due to denser and complex architectures. This selection process was instrumental in constructing an Ensemble-CAM framework composed of classifiers that not only excel in image-level classification but also in generating precise heatmaps for disease localization.

The experimental iterations for given datasets with specified hyperparameters concluded on DenseNet169, DenseNet121, InceptionResnetV2, and Xception as detailed in Table 4. These models, particularly the DenseNet architectures, excelled in localizing cardiomegaly within the Chest-Xray14 dataset and pneumonia in the RSNA dataset, while InceptionResnetV2 demonstrated exceptional precision across multiple conditions. Notably, for COVID-19 detection, DenseNet121 and InceptionResnetV2 demonstrated high accuracy and precision, highlighting their capacity for reliable pattern identification.

**Table 4:** Performance of classifiers on given datasets.

**Target class**	**Classifier**	**Acc**	**Recall**	**Precision**
Cardiomegaly (Chest-Xray14)	DenseNet169	0.95	0.92	0.90
	DenseNet121	0.94	0.91	0.89
	InceptionResnetV2	0.96	0.95	0.94
Pneumonia (RSNA)	DenseNet169	0.97	0.93	0.88
	Xception	0.93	0.93	0.90
	InceptionResnetV2	0.93	0.90	0.87
COVID-19 (COVID-19)	DenseNet121	0.97	0.95	0.95
	InceptionResnetV2	0.98	0.96	0.97
	Xception	0.97	0.92	0.94

The classifiers ultimately incorporated into Ensemble-CAM were deliberately chosen to strike an optimal balance between localization performance and computational demand. While the selected models–DenseNet169, DenseNet121, InceptionResnetV2, and Xception–require considerable computational resources due to their complexity, they also significantly enhance localization accuracy. This is essential for clinical applications where diagnostic precision is paramount. The selection process prioritized models that brought substantial improvements in localization accuracy without disproportionately increasing computational costs. This ensures that Ensemble-CAM delivers a high diagnostic value while remaining practical for use in diverse clinical environments, even where computational resources may be limited.

Finally, the chosen classifiers for Ensemble-CAM were carefully picked to ensure a good balance between accurate disease localization and the amount of computational power needed. These models do require more computational resources, but they provide better accuracy in pinpointing diseases on chest X-ray images. The decision to use these models was based on their ability to give clearer results for diagnosis without needing an unreasonable amount of computing power, making Ensemble-CAM a practical option for medical settings with varying levels of available technology.

### 3.4 Application of CAM

Ensemble-CAM utilizes class activation mapping techniques for achieving two objectives: (1) to generate heatmaps that make the outcome interpretable and (2) to extract spatial information for the localization task. While employing the CAM technique, the design goal was to avoid model alteration, re-training, and better visibility of detected objects. Three variants of CAM have been considered in the ablation study, namely Vanilla CAM (Definition 1), Grad-CAM (Definition 2), and Grad-CAM++ (Definition 3). Two limitations were identified in Vanilla CAM for the proposed framework, i.e., coarse visuals on heatmap image and model alteration with a global average pooling layer. To address these challenges, Grad-CAM (Selvaraju and Batra, 2020) was evaluated next as it offers better interpretability without trading-off the model structure and performance. Grad-CAM extracts a raw feature map during the forward propagation. This tensor is backpropagated to the desired rectified convolutional feature maps. This collectively computes the coarse Grad-CAM localization which explains where the model must look to make the specific decision. During experiments on X-ray images, Grad-CAM's ability to properly localize areas of interest was observed decreasing for multiple occurrences of the same class. The main reason for this decrease is emphasizing the global information that local differences are vanished in it. This impact has been minimized in Grad-CAM++ (Chattopadhay et al., 2018) which enhances the output map for the multiple occurrences of the same object in a single image. Specifically, it emphasizes the positive influences of neurons by considering higher-order derivatives.

**Notation**. Let us declare a convolutional neural network as *Y* = *f*(*X*), such that input *X* ∈ ℝ^*d*^ and output *Y* as a probability distribution. We define *Y*^*c*^ as the probability of being classified as class *c*. For a specified layer *l*, let *A*_*l*_ refer to the activation of layer *l*. Specifically, if *l* has been selected as a convolution layer, then $A_{l}^{k}$ denotes the activation for the *k*-th channel. This also denotes the weight of the *k*-th neuron at layer *l* which connects two layers *l* and *l*+1 as *W*_*i*+*l*+1_.

**Definition 1 (Class Activation Map)**. Using the defined notation, consider a model *f* consists of a global pooling layer *l* that takes the output from the last convolutional layer *l* − 1 and feeds the pooled activation to a fully connected layer *l* + 1 for classification. For a class of interest *c*, $L_{\text{CAM}}^{c}$ can be defined in Equation 6 as:


(6) $$\begin{array}{l} L_{\text{CAM}}^{c}=\text{ReLU}\left(\underset{k}{\sum }a_{k}^{c}A_{l-1}^{k}\right) \end{array}$$

$\begin{array}{ll} \text{where:} & a_{k}^{c}=W_{l,l+1}^{c}\left[k\right] \end{array}$

$\begin{array}{l} W_{l,l+1}^{c}\left[k\right] \end{array}$ is the weight for the *k*-th neuron after global pooling at layer l.

**Definition 2 (Grad-CAM)**. Using the stated notation, suppose a model *f* and class of interest *c*, Grad-CAM is defined in Equation 7 as:


(7) $$\begin{array}{l} L_{\text{CAM}}^{c}=\text{ReLU}\left(\underset{k}{\sum }a_{k}^{c}A_{l-1}^{k}\right) \end{array}$$

where:


$$\begin{array}{ll} a_{k}^{c} & =\text{GP}\left(\frac{\partial Y^{c}}{\partial A_{l}^{k}}\right)\\ \text{GP}\left(\;\right) & \text{denoted the global pooling operation.} \end{array}$$

**Definition 3 (Grad-CAM++)**. Using the stated notation, suppose a model *f* and class of interest *c*, Grad-CAM++ is defined in Equation 8 as:


(8) $$\begin{array}{l} L_{\text{gradCAM++}}^{c}=\text{ReLU}\left(\underset{k}{\sum }a_{k}^{c}A_{l-1}^{k}\right) \end{array}$$

where:


$$\begin{array}{ll} a_{k}^{c} & =\frac{1}{Z}\underset{i}{\sum }\underset{m}{\sum }\left(\frac{\partial Y^{c}}{\partial A_{l}^{k}}\right)\\ Z & \text{is a constant that refers to the number of pixels in the}\\ \text{activation map.} \end{array}$$

### 3.5 Ensemble-CAM using interpretable features

The input image consists of three types of features such as (1) noise, (2) relevant, and (3) salient features. Noise induces distraction in the classification task and subject to be removed by techniques such as Gaussian blur, median filtering, and various filters. The relevant features are referred to the domain of interest which is to identify the legitimate chest X-ray (CXR) image with the frontal view. The salient features are class-specific sub-part of the relevant features.

In this study, CNN models have been targeted during the classification task for extracting salient features using the class activation mapping technique. As explained in section D, CAM identifies parts of the image that contribute most to the target class. The feature interpretability of a CAM arises from the fact that it provides a visual representation of the CNN model's understanding of the input image features that are important for the classification decision. The heatmap generated by the CAM highlights the regions of the image that are most relevant for the CNN model's prediction and can be used to identify the key features that distinguish between different classes. This provides valuable insights into the decision-making process of the CNN model and can help to identify which image features are most important for making a diagnosis.

Ensemble-CAM offers a fusion scheme to highlight prominent sub-regions in the X-ray image. It consolidates activation maps that have been generated by more than one image classifiers in the heatmap format. The resultant heatmaps are intersected by high confidence function. Formally stating:

**Definition 4 (Ensemble-CAM)**. Suppose ensemble learning as a function *g* such that it produces a set of *n* number of heatmaps *H* (Equation 9); through models *M*; for a given input image *x*:


(9) $$\begin{array}{l} H=g\left(M\left(x\right)\right) \end{array}$$

where:

- *g*() symbolizes as ensembled function.
- *M*() refers to set of models; *m*_1_, *m*_2_, ......., *m*_*n*_ that predicts class *c*.
- *c* implies either the user-defined input that explicitly refers to a class or the maximum occurrence of a predicted class.
- *H* denotes the set generated heatmaps $\left\{h_{m_{1}}^{c},h_{m_{2}}^{c},......,h_{m_{n}}^{c}\right\}$.

Then, Ensemble-CAM is defined in Equation 10 as the intersection of *H* such that


(10) $$\begin{array}{l} L_{\text{ensembleCAM}}^{c}=h_{m_{1}}^{c}\cap h_{m_{2}}^{c}\cap \ldots \cap h_{m_{n}}^{c} \end{array}$$

The proposed model is also expressed in Algorithm 1. First, input radiograph *x* is classified by all the given image classifiers *m*_1_, *m*_1_, ....., *m*_*n*_ to predict class values ĉ_1_, ĉ_2_, ....., ĉ_*m*_. The majority of class predicted value determines final predicted class such that *c*←argmax([ĉ_1_, ĉ_2_, ......, ĉ_*m*_]) . The final class value *c* along with original radiograph *x* are provided to cam function (Grad-CAM++) as an input. Each classifier generates a heatmap image as $\left\{h_{m_{1}}^{c},h_{m_{2}}^{c},......,h,h_{m_{n}}^{c}\right\}$.

**Algorithm 1:** Ensemble-CAM.

Require: Image *X*∈*R*^*d*^, target class *c*, models = [*m*1, *m*2, *m*3], cam=[gradcam]
Ensure: Heatmap *H*, predicted class *c*, Bounding box (x, y, width, height)
1: number_of_models ← count(models)
2: Clist ←[]
3: for i ← 1 to number_of_models **do**
4: *mi*← models[i]
5: *ci*← mi.predict_class(X)
6: push(*ci*, Clist)
7: end **for**
8: if c = null **then**
9: c ← argmax (Clist)
10: end **if**
11: for i ← 1 to number_of_models **do**
12: *mi*← models[i]
13: *H*_*mi*_← mi.predict_map(X,c)
14: gray ← extract_channel(*H*_*mi*_, 'red')
15: ret, thresh = threshold(gray,127,255,0)
16: contours, hierarchy = findContours(thresh)
17: rect = minAreaRect(contours))
18: end **for**

The aim of Ensemble-CAM is to increase the probability of true positive inferences at the pixel level by reducing noise and irrelevant regions. Hence, it produces more reliable spatial regions within the X-ray image. This study demonstrates the outcome of Ensemble-CAM for estimating bounding boxes without being trained on bounding box annotations (x, y, w, h). As discussed previously, class of a disorder at the image-level is predicted by three models independently. The top-ranking class is declared final automatically by the maximum voting scheme. Any class of interest can also be selected manually, if needed, for analysis. Next, Grad-CAM++ generates class-oriented heatmap in the jet-colormap scheme and the red channel is sliced for corresponding visual semantics.

## 4 Results and discussion

The aim of Ensemble-CAM is to offer reliable and interpretable localization details without being explicitly trained on localization data. It supports the adaptation of the existing state-of-the-art image classification models and CAM function that require no alteration in the architecture. In addition to extending image classifier capabilities for the localization task, the framework presents the outcome in an explainable layout.

In this study, multiple deep learning models have been trained on three datasets of X-ray images (see Table 4).

During the testing phase, it was observed that different image classifiers may not always predict the same class for the same input X-ray image. This induces the unreliability aspect of employing the single model for diagnosis task. Such behavior validates the adaptation of assembling approach to overcome the probability of false predictions. Subsequently, the classification task with ensemble learning improved the overall performance.

Alongside classification, the also expect the model to justify the outcome. In traditional machine learning models such as decision trees, one could find such justifications in if-then hierarchies. Deep learning models are considered too opaque for if-then justifications in the image classification task. One alternative for such reasoning is supervised learning localization where areas-of-interest are highlighted either by masking or bounding boxing. This option is depended on rich annotated data that are difficult to acquire in higher quantity with adequate quality. Another alternative is to leverage the classification knowledge for localization as weakly supervised learning approach. We opted later option with class activation mapping techniques to achieve two objectives, i.e., localization and interpretation. Among CAM variants, Grad-CAM++ was found best suited for its ability to be adapted without altering the model while extracting finer localization information in case of multiple instances. Equally, it has also been found useful visual explainer for interpreting its outcome intuitively when heatmap images were generated.

As discussed, *n* numbers of classifiers produce *n* numbers of predictions in ensemble models. An aggregating function is therefore required to draw a single conclusion. Likewise, localization task also follows the same process, i.e., *n* classifiers produce *n* heatmaps which further require aggregation. We employed maximum voting function to achieve the confident value for final localization. This function has been applied at pixel level where maximum intersection occurs. Finally, minimum area rectangle has been formed from the qualified pixels' left-top and right-bottom coordinates.

This study demonstrates the performance of the proposed model on three chest X-ray datasets for detecting three different pneumonia, COVID-19, and cardiomegaly. The RSNA pneumonia detection dataset has been used for training and validation. Although the dataset offers ground truth labels, they were not used to follow a weakly supervised approach. Image classifiers were trained on images using image-level class labels. Once trained, images from the test set were asked to classify and localize. The results were compared with the ground truth values to calculate the Jaccard index. The same strategy has been followed for detection of cardiomegaly using the Chest-Xray14 dataset. As both the datasets possess bounding-box level ground truth labels, the Jaccard index was calculated. For detecting COVID-19, the model has been trained only on images with class labels. However, the Jaccard index has not been computed as bounding box annotations for this dataset are not available.

**Figure 3:**
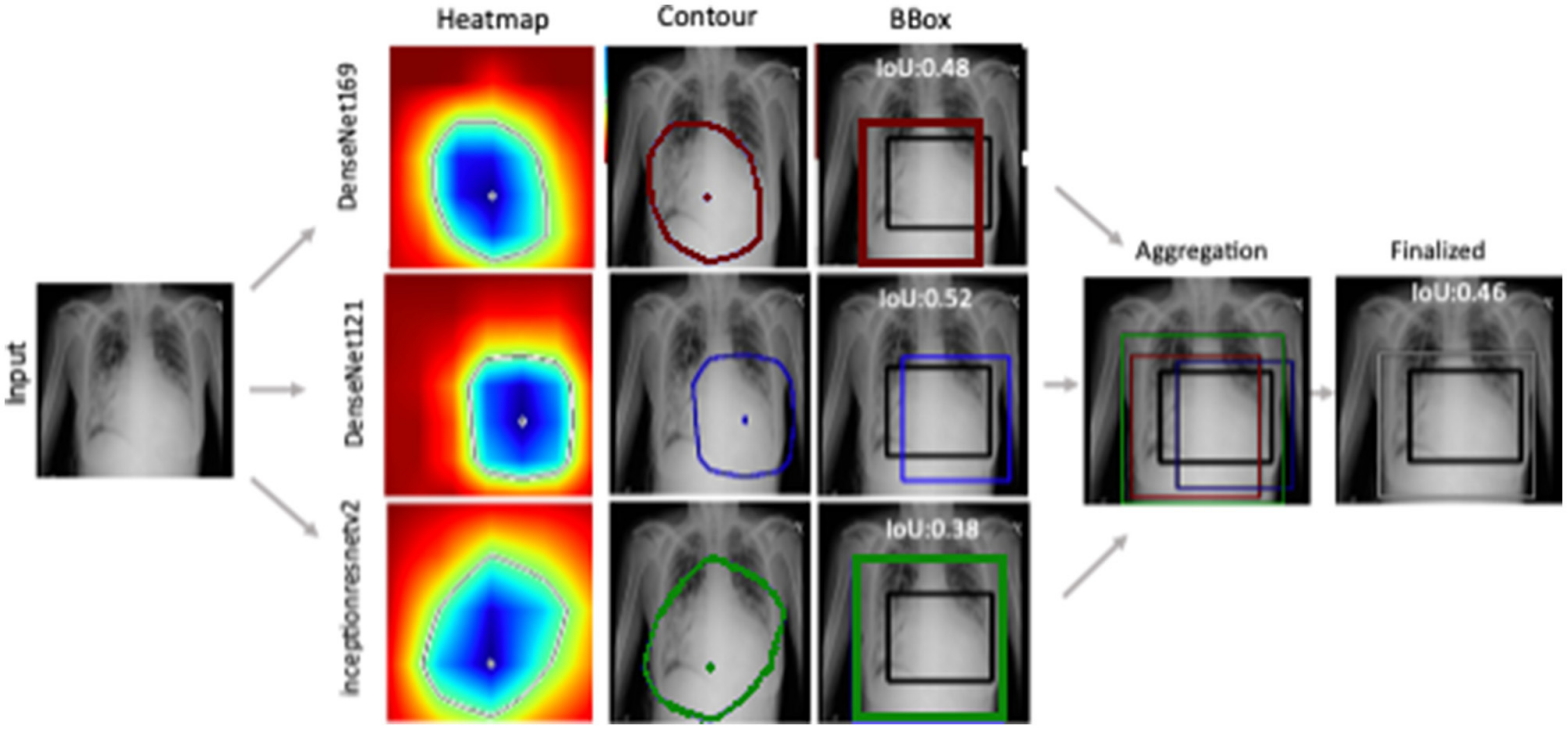
Ensemble-CAM generates localization information from image level class labels while making the process interpretable. Reference to labeled data, IoU has been computed for quantification of the result.

Figure 3 shows the generated heatmaps in the BGR color scheme referring to the intensity of activation from the highest to lowest. The green color serves as the border between the highest (blue) and lowest values (red). To form an estimated mask, a contour is drawn by connecting the green pixels as convex hull. The resultant polygon is served as the mask when filled with binary 1 while marking the rest as binary 0. Though the mask offers better localization, we proceeded to generate bounding boxes. The first reason is the demonstration of model capability for predicting bounding box. The second reason is to evaluate the localization performance with available annotation. The example of ground truth valued BBox is shown in Figure 3 in black color as a reference while the computed Jaccard index is displayed on the top.

**Figure 4:**
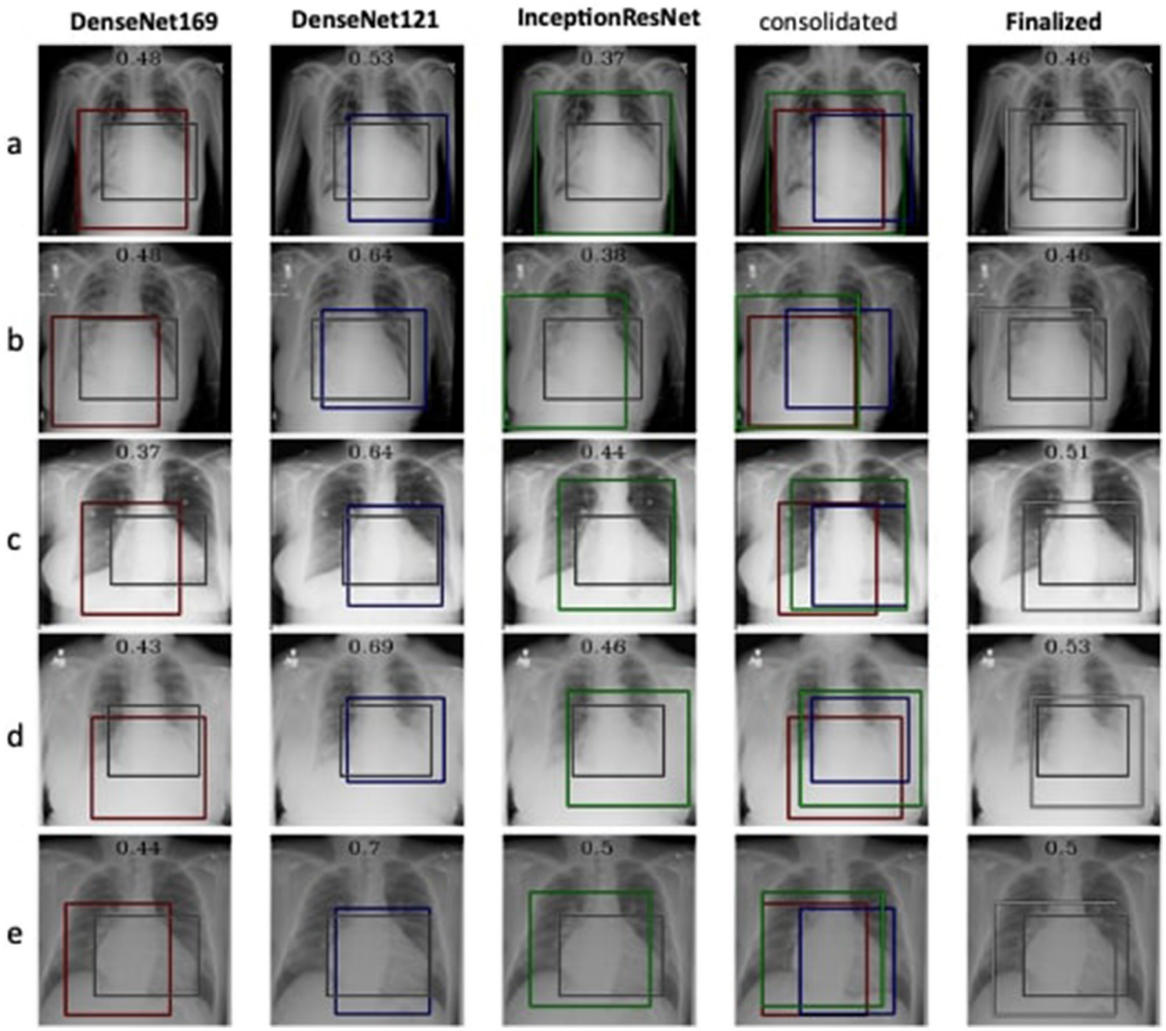
Estimation of bounding box annotation for cardiomegaly localization quantified by IoU scores.

The detection and localization of cardiomegaly are shown in Figure 4 for few samples. The model consists of three classifiers, namely DenseNet169, DenseNet121, and InceptionResNet. The size of BBox among these classifiers can be observed as the first visual difference. DenseNet121 and DenseNet169 belong to the same family of architectures and form smaller and medium BBoxes respectively. InceptionResNet comparatively creates larger BBoxes with least accuracy in the collection. The consolidation step aggregates all the three BBoxes into single BBox to form a conclusive outcome. As the dataset is furnished with a small set of ground truth BBox annotations, quantitative results have also been computed using the Jaccard index. Table 5 presents the computed values for the listed sample images. The same values can also be visualized at the center-top of each image under the classifier column. The performance varies from image to image among the classifiers. In the case of cardiomegaly, DenseNet121 constantly outperforms all radiographs while DenseNet169 and InceptionResNetV2 alternate for second place. This also ensembled outcomes to form comparatively coarse IoU because it considers cumulative intersections. For such configuration, a practitioner can give more value to the best classifier's predictions. However, there exist scenarios where single classifier may not always point to the right locations. Such scenarios have been demonstrated for the detection of pneumonia in the next model.

**Table 5:** IoU detection score for the detection of cardiomegaly (ref. Figure 4).

**Input instance**	**DenseNet 169**	**DenseNet 121**	**Inception ResNetv2**	**Finalized**
a	0.48	0.53	0.37	0.46
b	0.48	0.64	0.38	0.46
c	0.37	0.64	0.44	0.51
d	0.43	0.69	0.46	0.53
e	0.44	0.70	0.50	0.50

**Figure 5:**
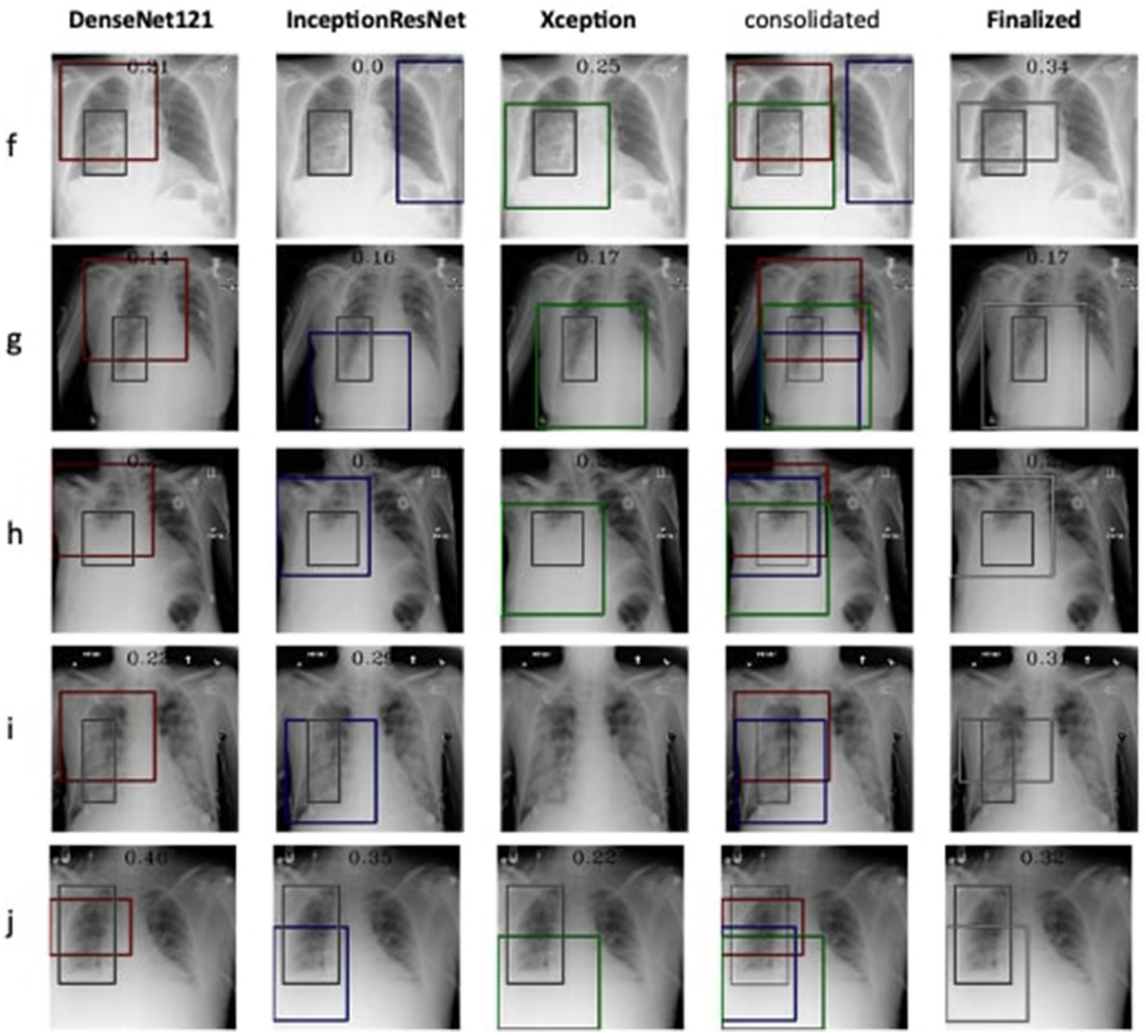
Estimation of bounding box annotation for Pneumonia localization quantified by IoU scores.

**Figure 6:**
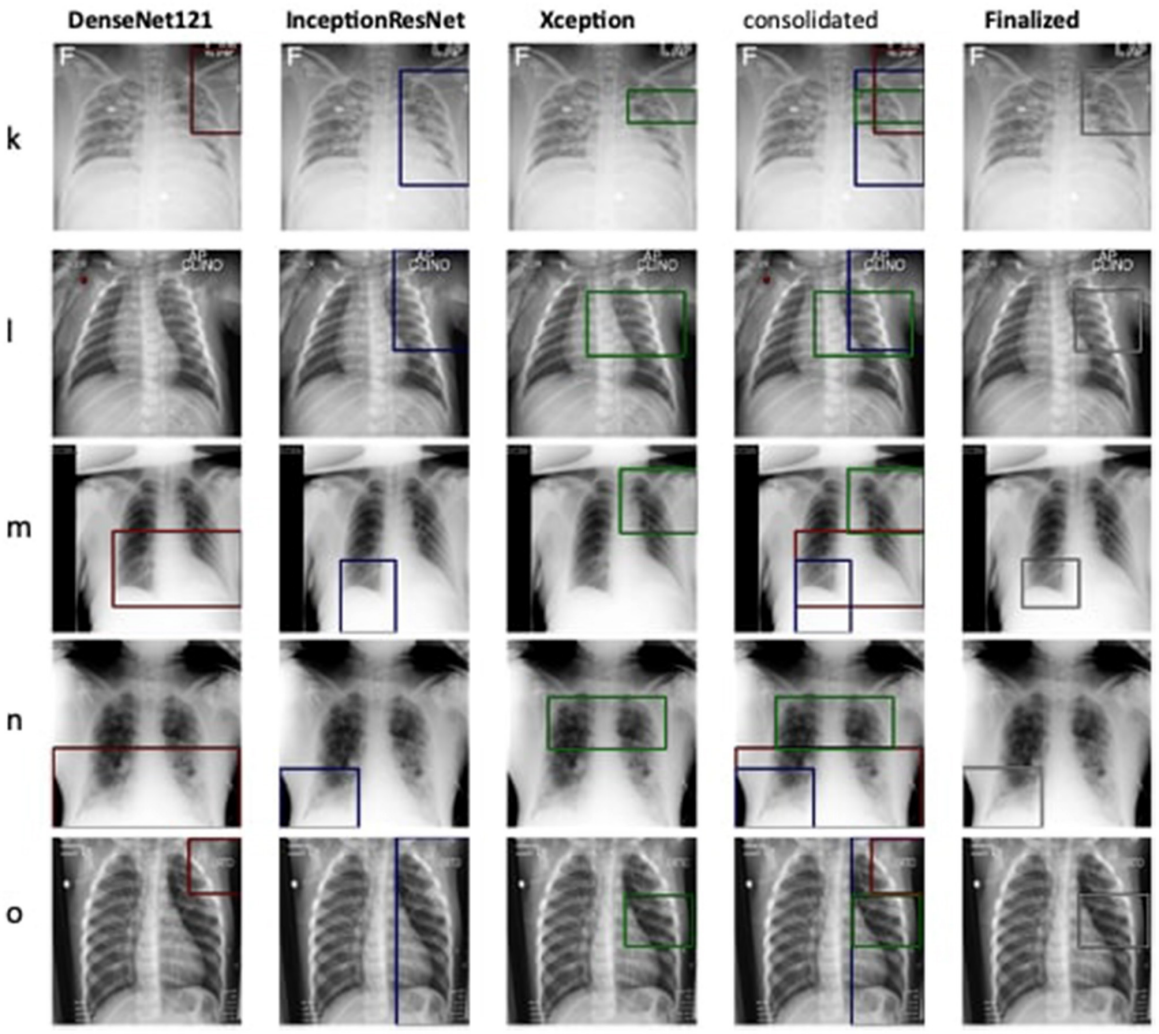
Qualitative illustration of predicting bounding boxes for COVID-19 cases.

Therefore, the next better classifier was employed, which belongs to the InceptionResNet family architecturally. Ensemble-CAM is agile enough to replace any of its components when required without any further alteration in the framework. In this instance of model, it can be observed that the performance of pneumonia detection is not good enough compared to the instance of cardiomegaly. The reason can be traced out by observing the generated heatmaps on different radiographs. For instance, we found that Dense-Net169 is consistently highlighting the lower part of radiographs for its opacity to declare it pneumonia. Once found the issue with learning, we have options to either fine-tune it by changing the hyperparameters, perform further training with filtered data, or combine the bast of both. Nevertheless, we replaced it with another successor because of its availability reason. Regarding the model performance, none of the sub-models show consistency in producing finer localization for all given radiographs. This can be observed quantitatively via Table 6. Xception shows the best IoU on input f and h and least for i and j. likewise, DenseNet121's best IoU is for j while InceptionResNetV2 ranks first for h. Visual conformance of the stated scenario is illustrated in Figure 5 where the black outlining box has been referred as ground truth for the generated boxes. This creates the need for collecting the proposals from all classifiers and form a one that honor their mutu-al/intersected arguments. The last use-case has been demonstrated in Figure 6 for the fact that some datasets may not have any bounding box information even for test purposes and still detection task is demanded. This illustrates the application of proposed model for the detection of COVID-19 symptoms. The associated dataset does not provide ground truth values; therefore, quantitative results were not computed on the Jaccard index. For visual analysis, the model is supposed to highlight ground-glass opacity in the lungs area. Since pneumonia and COVID-19 share similar characteristics, we adapted pneumonia detecting classifiers for COVID-19. The combined results of pneumonia and COVID-19 show high variance in performance. They are not fully consistent on mutual agreement and so result in poor performance.

**Table 6:** IoU detection score for the detection of pneumonia (ref. Figure 5).

**Input Instance**	**DenseNet 121**	**Inception ResnetV2**	**Xception**	**Finalized**
f	0.21	0.0	0.25	0.34
g	0.14	0.16	0.17	0.17
h	0.23	0.30	0.24	0.27
i	0.22	0.29	0.0	0.31
j	0.46	0.35	0.22	0.32

The proposed study differs in localization techniques such as YOLO, SSD, etc. in terms of supervision, i.e., strong vs. weak. It induces explainability while extracting interpretable features for localization task using CAM. To enhance the reliability on prediction, it offers ensembled strategies for classifiers and localizers without alteration in the base models. The performance of IoU can be discussed in two perspectives. In comparison to strong-supervised approaches, they may not touch the benchmark. However, they are highly dependent on spatial-annotated data. To overcome this dependency, weak supervised learning offers localization as an alternative approach with lower IoU. They only require image-level labels during training. For Ensemble-CAM, the cumulative results of Ensemble-CAM for given datasets show promising results in localizing abnormalities within chest radiographs. This framework is based on loosely coupled components that are replaceable and extendable to tune up the overall performance. Moreover, it offers interpretability for debugging the training deficiencies as well as justification at the prediction stage. Leveraging its interpretability features, the model also exhibits favorable results for estimation of mask and bounding box annotations by getting trained on only class labels. Taking these capabilities into account, Ensemble-CAM can play a vital role in assisting reliable diagnosis in clinical practice. Although it eliminates the need for strong annotation for training, it requires more computational resource for training and for prediction.

To further advance the capabilities of our Ensemble-CAM framework, we are committed to addressing the current limitations and exploring new dimensions in thoracic disease analysis. Future efforts will include the adoption of additional quantitative metrics, such as the DICE coefficient and Precision, to enhance the evaluation of localization and detection accuracy. These metrics will provide deeper insights into the model's performance and its effectiveness in clinical settings. Moreover, we are planning to improve the system's architecture by integrating unified classifiers designed to process a broader spectrum of thoracic diseases. This development aims to achieve a more comprehensive and efficient diagnostic tool, capable of providing robust analyses from chest X-ray images. By pursuing these enhancements, we intend to not only refine the diagnostic accuracy of our system but also to broaden the scope of its applicability in medical imaging, ensuring that our research contributes continuously to the evolving field of AI in healthcare.

## 5 Conclusion

The diagnosis of thoracic diseases using chest X-ray images is a critical and sensitive area. It has many risks for incorrect conclusions due to workload, skillset, and other subjective errors. Assisting medical professionals with AI powered computer aided systems using deep learning face multiple challenges. This study focuses on the challenges of inadequate data and interpretable inferences for deep learning models and presents Ensemble-CAM. It has been formulated as a unified model that utilizes the existing classifiers and class activation mapping to detect and localize thoracic disease in chest X-ray images. Three independent experiments on respective chest X-ray datasets have been conducted. During the training phase, no localization details were considered to predict bounding boxes. The generated heatmaps were evaluated both visually and quantitively. In comparison to the existing standalone models, Ensemble-CAM carries the lowest risk of incorrect classification errors when it encounters noisy features in X-ray images. This enhances the overall confidence on deep learning models for clinical practice. The theoretical contribution of Ensemble-CAM is envisioned in explainable AI and weak supervised learning spaces. This further contributes to the elevation of confidence on deep learning models to be employed in medical practice. In future studies, we aim to broaden the research scope by incorporating more image classifiers, exploring different CAM variants, and refining ensemble strategies. These enhancements are expected to provide deeper insights and higher accuracy, further leveraging the potential of AI in medical imaging and continuing the evolution of reliable, interpretable diagnostic tools for clinical practice. In future studies, we will enhance Ensemble-CAM by adding metrics, such as DICE and Precision, and developing unified classifiers. These steps aim to improve accuracy and broaden clinical use, contributing further to medical imaging and AI.

## Data availability statement

The original contributions presented in the study are included in the article, further inquiries can be directed to the corresponding author.

## Author contributions

MA: Conceptualization, Data curation, Formal analysis, Methodology, Software, Validation, Visualization, Writing – original draft. MJ: Conceptualization, Formal analysis, Investigation, Methodology, Project administration, Resources, Software, Supervision, Validation, Visualization, Writing – review & editing.
